# Routine use of DHIS2 data: a scoping review

**DOI:** 10.1186/s12913-022-08598-8

**Published:** 2022-10-06

**Authors:** Elaine Byrne, Johan Ivar Sæbø

**Affiliations:** HISP Centre and Department of Informatics, University of Oslo, Gaustadalléen 30, N-0373 Oslo, Norway

**Keywords:** Routine data use, DHIS2, Scoping review, Health information system

## Abstract

**Background:**

In regard to health service planning and delivery, the use of information at different levels in the health system is vital, ranging from the influencing of policy to the programming of action to the ensuring of evidence-informed practices. However, neither ownership of, nor access to, good quality data guarantees actual use of these data. For information to be used, relevant data need to be collected, processed and analysed in an accessible format. This problem of underused data, and indeed the absence of data use entirely, is widespread and has been evident for decades.

The DHIS2 software platform supports routine health management for an estimated 2.4 billion people, in over 70 countries worldwide. It is by far the largest and most widespread software for this purpose and adopts a holistic, socio-technical approach to development and implementation. Given this approach, and the rapid and extensive scaling of DHIS2, we questioned whether or not there has been a parallel increase in the scaling of improved information use. To date, there has been no rigorous review of the documentation on how exactly DHIS2 data is routinely being used for decision-making and subsequent programming of action. This scoping review addresses this review gap.

**Methods:**

The five-stage approach of Arksey and O’Malley progressed by Levac et al. and Peters was followed. Three databases (PubMed, Web of Science and Embase) were searched, along with relevant conference proceedings and postgraduate theses. In total, over 500 documents were reviewed and data from 19 documents were extracted.

**Results:**

Overall, DHIS2 data are being used but there are few detailed descriptions of this usage in peer reviewed or grey literature. We find that, commonly, there exists a centralised versus decentralised pattern of use in terms of access to data and the reporting of data ‘up’ in the system. We also find that the different conceptualisations of data use and how data use is conceptualised are not made explicit.

**Conclusions:**

We conclude with some suggestions for a way forward, namely: i) the need to document in more detail and share how data are being used, ii) the need to investigate how data were created and who uses such data, iii) the need to design systems based on work practices, and in tandem develop and promote forums in which ‘conversations’ around data can take place.

**Supplementary Information:**

The online version contains supplementary material available at 10.1186/s12913-022-08598-8.

## Background

In regard to health service planning and delivery, the use of information at different levels in the health system is vital, ranging from the influencing of policy to the programming of action to the ensuring of evidence-informed practices [1–3]. There have been global calls to action, consortia, and frameworks to support information within the remit of health systems strengthening, for example: the Paris Declaration of 2005 and the establishment of Health Metrics Network the same year; World Health Organization’s Framework for Action; the focus on Strengthening Health Systems to Improve Outcomes in 2007; and the U.S. Global Health Initiative in 2011 [4]. The rationale for these commitments is that better quality data, that are both relevant and comprehensive, will increase use of these data in action and decision-making and ultimately improve health service delivery and health outcomes. However, neither ownership of, nor access to, good quality data guarantees actual use of these data [5–7]. To ensure information use, relevant data need to be collected, processed and analysed in an accessible format [6]. This problem of underused data, and indeed the absence of data use entirely, is widespread and has been evident for decades [8–11].

The existing literature reviews that examined data use have focused more on challenges faced rather than sharing of solutions and identifying ways to address these challenges. For example, Lemma et al. [12] in their 2020 scoping review of interventions that aimed to improve data quality and its use in routine health information systems in Low and Middle Income Countries (LMICs) classified challenges regarding data quality and its use in relation to staff, resources, or infrastructural factors. In a systematic literature review Wickremasinghe et al. [13] examined how district administrators and health managers in LMICs used health data to make decisions and found that there was a limited range of processes documented on the use of data for decision-making at district level.

A partial explanation for limited data use is that more emphasis has been placed on data collection in LMICs than on data-use itself, with evaluations of these systems focusing more on statistical data processes and data quality, and less on how data are assimilated into practice [14]. Other studies attribute limited data use to suboptimal quality of data generated by the routine health information systems, and to an absence of a culture of information-use [15–17]. This suboptimal quality of data may be due to unintended mistakes or deliberate misreporting,^1^ but other factors contributing to poor quality can include under-reporting or no reporting at all due to time pressures, lack of motivation, too many forms to complete and a lack of understanding of the importance of data [7]. Additionally, apart from poor data quality, there remains the possibility that no standardized process governing the usage of data exists [18, 19]. So, despite these reviews and studies exploring data use, there remains limited knowledge or understanding of how data are being used or which data and processes are involved.

## Data use

Data use is not easy to define as both ‘data’ and ‘use’ can be conceptualised in many different ways. A Delphi study of information scientists by Zins (2007) yielded more than 40 different definitions of data, while Checkland and Holwell (1998) revealed 7 different definitions in Information Systems textbooks [quoted in 20]. Jones [20] describes a number of assumptions that are made around data that we should question: that all data are equal, that data represent a reality independent of themselves, that data exist independently of their use, that data form the foundation on which our understanding is built, and that data represent the world objectively. When we question these assumptions, we realise that data do not necessarily report reality, that data are not recorded in a vacuum but reflect a particular worldview, that data are interpreted and may be non-empirical, and that data may vary in their perceived value and quality. Once questioned in this manner we need to distinguish, as Jones [20] suggests, between “data in principle” (as they are recorded), and the “data in practice” (as they are used). In this review we are concerned with “data in practice”.

There are also different conceptualisations and hence definitions of data use. Manuals and reports on DHIS2 itself have referred to the information cycle as illustrative of the stages required before information gets used (collection; processing; analysis; presentation; interpretation and use). In this sense data use is defined as the last step in a process and fits the definition of use by Foreit et al.: “Decision makers and stakeholders explicitly consider information in one or more steps in the process of policy making, program planning and management, or service provision, even if the final decision or actions are not based on that information” ([21], p.5).

Similarly, Nutley interprets data use in decision-making “.. as the analysis, synthesis, interpretation, and review of data for data-informed decision-making processes, regardless of the source of data. ‘Data-informed decision making,’ then, refers to the proactive and interactive processes … that consider data during program monitoring, review, planning, and improvement; advocacy; and policy development and review” ([4], p.2). Nutley concludes that “… it is clear that data use goes beyond filling out data reporting forms at the various levels of a national health information system and the passive dissemination of reports and information products.” ([4], p.2). However, Nutley proceeds to extend this conceptualisation to the purpose of use.

Nutley [4] categorises data use in terms of data and information regularly demanded, analysed, synthesised, reviewed and used in: (i) program review and planning, (ii) advocacy and policy development, and (iii) decision-making processes. Nutley doesn’t define each of these categories but classifies all three as the long-term outcomes of the use of data.

In addition to the aspects of process and purpose, the Health Metrics Network framework [22] can be applied to illustrate variance in content, reflecting the diversity of uses and users and involving the wider community such as civil society. Data use in the Health Metrics Network framework’s definition involves varied levels of data granularity and a wide range of information products.

We return to this conceptualisation and definition of data use in subsequent discussion but data use in this review was explored in a way that transcends mere data collection, form filling and the passive production and dissemination of reports or products. We therefore examined documents that either covered the process, the purpose and/or the content governing the use data from DHIS2 – what Jones’ [20] would distinguish as the use of ‘data in practice’.

## District Health Information Software 2 (DHIS2)

Because DHIS2 is a prominent Health Management Information Systems (HMIS) platform in LMICs, this study investigated the use of DHIS2 data in these countries. Typically, DHIS2 is used as the national health information system for: data management and analysis purposes, health program monitoring and evaluation, facility registries and service availability mapping, logistics management and various community-based services such as mobile tracking of pregnant mothers in rural areas. Alongside increased support and adoption of DHIS2, the strengthening of HMIS has been facilitated by both increased commitment and investment. The DHIS2 software platform has, over the last decade, witnessed tremendous adoption, and now supports routine health management for an estimated 2.4 billion people^2^. It is used in over 70 countries and is by far the largest and most widespread dedicated health management software. So, the question is this: has the rapid and extensive scaling of DHIS2 been matched by a corresponding increase in the scaling of improved data use?

The Health Information Systems Programme (HISP) is a global action research network to support DHIS2 implementation, to facilitate local customisation and configuration, to offer in-country and regional training, and to promote DHIS2 as a global public good. HISP University of Oslo collaborates with a global network of HISP Groups in 17 countries in Asia, Africa, and the Americas. Walsham [7] notes that improved information necessitates an approach that combines the three elements of ‘software philosophy, educating people and changing institutions’ and cites the work on the HISP [23, 24] as a programme that addresses information systems from all three perspectives. However, despite this, ‘we still see only limited evidence as to how health information systems have contributed to improved health outcomes, and to advancing the state of the poor in developing countries.’ ([7], p.196). So, if DHIS2 is the largest global routine HMIS in LMICs and adopts a holistic socio-technical approach to development and implementation, and yet despite these data is still not being used for information, action taking and/or decision making then we are duty bound to explore why this is the case. However, there has been no rigorous review conducted on how DHIS2 data are being used despite the tremendous success recorded in scaling, implementation, and improvements in data quality and data access.

There are few reviews on data use and, more particularly, on data use in relation to DHIS2. In a review of the utilisation of DHIS2 data in decision-making (at the district, sub-district, and community levels in selected districts of the Brong Ahafo region in Ghana), Odei-Lartey et al. [25] explored the various facilities’ routine meetings in search of evidence of decision-making. Though they concluded that the use of DHIS2 data to inform decisions was suboptimal they also discovered that data were being used in regard to discussions about the DHIS2 platform itself, that findings from DHIS2 data informed action-oriented decisions in addition to actions taken to promote the usage of the DHIS2 platform. The 4 categories of action-oriented decisions were: i. performance recognition and role/responsibility revision, ii. shifting/mobilization of resources, iii. advocacy for more resources and iv. formation/revision of policies/strategies. A recent literature review of DHIS2 [26] explored the strengths and operational challenges in the technical and functional aspects of DHIS2 in 11 countries but did not focus on data use. Additionally, these reviews focus on peer-reviewed literature and thus exclude a large amount of grey literature such as conference papers and research theses in this area. Consequently, our review addresses this gap and focuses specifically on the documentation of routine use of the DHIS2 data for action and decision making.

## Methods

Scoping reviews have been used widely ‘to identify knowledge gaps, scope a body of literature, clarify concepts or to investigate research conduct’ [27]. They are useful ‘when a body of literature has not yet been comprehensively reviewed or exhibits a complex or heterogeneous nature not amenable to a more precise systematic review of the evidence’ ([28], p141). Scoping reviews can also document research that informs and addresses practice [29]. A scoping review does not include aggregation and synthesis of data nor does it include an assessment of the quality of the documents included [27].

Thus, a scoping review suits our review consideration, namely, to map how routine DHIS2 data use has been documented. Our objectives were to review the literature (peer reviewed and grey) regarding DHIS2 data use, and to categorise key examples of use of DHIS2 data. This scoping review included a review of peer reviewed literature, key journals and conferences, and theses produced within the HISP programme. The primary research question uses the PCC method where the population group are users of DHIS2 data; the concept is DHIS2 the software, and the context is LMIC health systems. Therefore, the review question is: ‘How are DHIS2 data being used for action and decision making within LMIC health systems?’ Sub-questions explored to address the primary research question also included:

1. In what areas is it reported that DHIS2 data are being used?
2. What are the reported examples of DHIS2 data being used for action and decision making?

The following databases were searched for peer reviewed literature: Pubmed, EMBASE and Web of Science, as these are deemed the most relevant for literature related to the topic (see search strategies in Additional file 1: Appendix 1). The time frame for the search extended from the date of publication of the first article in a given database to March 20^th^, 2021.

Due to language limitations, we included only English language articles. We hand searched (manually searched) reference lists of studies deemed to be highly relevant to the review question in order to identify other relevant studies. We sourced grey literature from the International Federation for Information Processing: Working Group 9.4 (IFIP 9.4) conferences (central as they were to HISP researchers’ ability to share their DHIS2 research), and from the Post Graduate (MSc and PhD) theses from the HISP in the Department of Informatics at the University of Oslo. A review of evaluations and assessments of DHIS2 internal to HISP was conducted as part of a separate study by the first author (EB) but this did not reveal any additional detailed examples of data use not previously included in other publicly available documents. These internal reports were not included as part of the scoping review, and consequently no ethical clearance was needed to conduct the review as all consulted material is publicly available.

Both authors (EB & JS) analysed the abstracts and full articles for review according to the inclusion or exclusion categories separately. Where there were conflicts, the authors met and resolved them. Colleagues from existing research and DHIS2 implementation groups within the department agreed to be included if a third opinion was needed, but most of the disagreements centred on ambiguity about the level of detail required for inclusion rather than whether or not articles met the inclusion/exclusion criteria. In these cases, the relevant articles were included in the full text review.

As noted, this review’s sole focus was DHIS2 (and previous versions of DHIS). Inclusion criteria demanded that research and conference articles were peer reviewed and described how the data from DHIS2 was being used for action / decision making OR that grey literature described how the data from DHIS2 was being used for action / decision making. Exclusion criteria included:

i) Articles that focused on use of data (for action or decision making) not from DHIS2
ii) Articles that evaluated or assessed the *needs* of the health system in relation to DHIS2, or the use of DHIS2 data
iii) Articles that described/evaluated quality of data only
iv) DHIS2 data used with other data sources with the purpose of validating or highlighting deficiencies of the datasets
v) Articles that solely described theoretical or conceptual frameworks that could improve DHIS2 data use
vi) Articles that solely described the analysis and products of data with no description as to how this analysis or these products were used
vii) Articles that mentioned data use but provided no examples of how it was used
viii) Non-English language studies

The five-stage approach of Arksey and O’Malley [29], progressed by Levac et al., [30] and culminating in the JBI Guidelines approach of Peters et al. [28] was followed. It included the following steps: definition and alignment of objective/s and question/s; development and alignment of inclusion criteria with the objective/s and question/s; description of the planned approach to evidence searching, selection, extraction, and charting; the final searching, selecting, charting, and summarising of evidence. The protocol was initially shared with the *Heritage Project*: *Designing for Data Use* (a research group with HISP at the University of Oslo) and its input was invited.

Duplicates were removed electronically in Covidence—a web-based software platform that supports all the steps of systematic literature reviews. Both authors independently screened titles and abstracts using Covidence for inclusion/exclusion. Disagreement between coders was resolved between team members, and even though, as mentioned, internal research groups were available for consultation this was not needed. For full article review both authors agreed on inclusion and exclusion independently and resolved any conflicts—again there was no need to bring in other groups as conflicts were easily resolved.

An extraction template was agreed upon and EB and JS extracted the full articles and grey material using this template. The data extraction template contained: author(s); year of publication; study title; journal/document source; study location; level of health system and health programme; study rationale; and description of use of data. Data was charted and exported from Covidence into Excel software. Standard descriptive information of included texts such as study site, year of publication, type of publication and health level and programme was conducted using this Excel spreadsheet. Study rationale and description of data from the charted data were subsequently categorised in relation to the focus of the study in terms of data use purpose, content, or process.

The findings from the scoping review were presented to the Paper Development Seminar Series at the Department of Informatics, University of Oslo and subsequently shared with other research groups and key individuals external to University of Oslo and their comments invited. This sharing of early drafts was for the purpose of validating the data that were included and providing an opportunity for colleagues to mention other articles or documents, especially grey literature, that we may have missed. The sharing also served to further discussion on what could be done to document use of DHIS2 data and the different conceptualisations of data use.

Both authors are currently part of HISP and by implication can be deemed ‘insiders’, but as noted in Byrne et al. [31] there are both advantages and disadvantages to this ‘insider’ versus ‘outsider’ status. A clear advantage in our case was the knowledge of the network, as well as the ability to identify who was involved in research and documentation of data use. The systematic approach of a scoping review coupled with the sharing of findings with key stakeholders have lent rigour to this review and contributed to a more collegiate interpretation of discovered data.

## Results

Findings are presented in two parts – a descriptive analysis of the texts included addressing sub-question 1, and categories of DHIS2 data use reported addressing sub-question 2.

### Description of texts included in the review

**Fig. 1:**
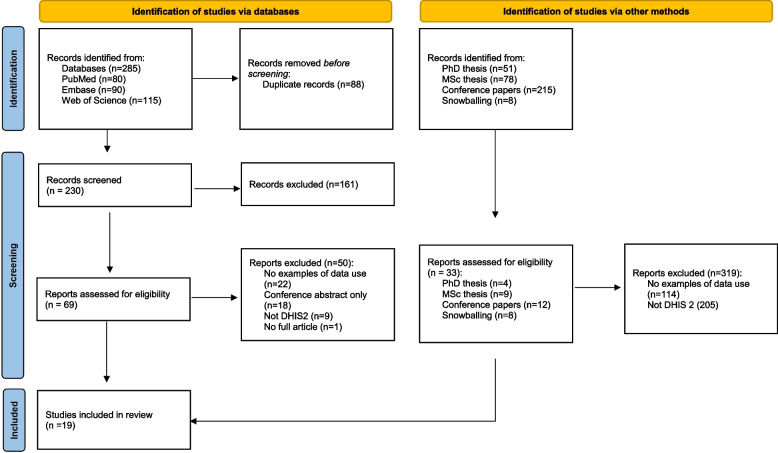
Preferred Reporting Items for Systematic Reviews and Meta-Analyses (PRISMA) for the scoping review. (*From:* Page MJ, McKenzie JE, Bossuyt PM, Boutron I, Hoffmann TC, Mulrow CD, et al. The PRISMA 2020 statement: an updated guideline for reporting systematic reviews. BMJ 2021;372:n71. https://doi.org/10.1136/bmj.n71. For more information, visit: http://www.prisma-statement.org/)

**Fig. 2:**
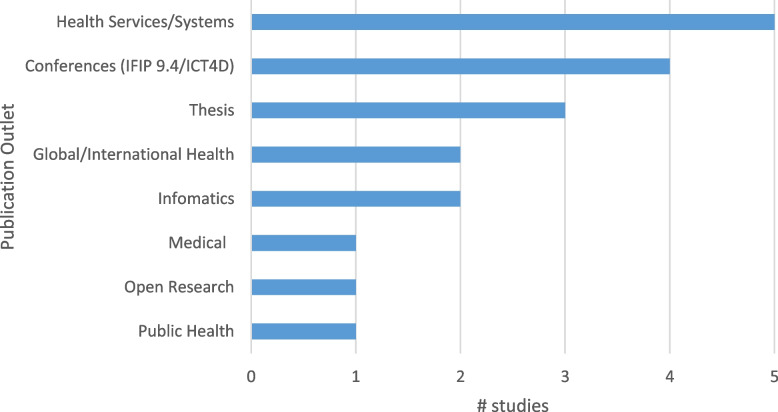
Publication outlets for studies included

A total of 19 documents were included – Fig. 1 illustrates the process by which the documents were included and excluded. Table 1 includes a brief description of the full text documents included. Included texts were mainly from the field of development informatics (the IFIP9.4 conference), from health services journals or as part of a thesis. Interestingly, only two of the articles were in informatics journals, perhaps indicating the focus on the design, implementation, and analytics in these journals and not on actions taken, or decisions made, based on collected data (see Fig. 2).

**Table 1:** Full texts included in review

Author(s)	Year	Title	Journal
Asah et al. [32]	2017	Challenges for Health Indicators in Developing Countries: Misconceptions and Lack of Population Data	14^th^ IFIP 9.4 WG Conference
Begum et al. [33]	2020	Perceptions and experiences with district health information system software to collect and utilize health data in Bangladesh: a qualitative exploratory study	BMC Health Services Research
Biemba et al. [34]	2017	A Mobile-Based Community Health Management Information System for Community Health Workers and Their Supervisors in 2 Districts of Zambia	Global Health: Science and Practice
Biswas [35]	2017	Shifting paradigm of maternal and perinatal death review system in Bangladesh: A real time approach to address sustainable developmental goal 3 by 2030	F1000Research
Braa et al. [9]	2012	Improving quality and use of data through data-use workshops: Zanzibar, United Republic of Tanzania	Bulletin of the World Health Organization
Chanyalew et al. [36]	2021	Routine health information system utilization for evidence-based decision making in Amhara national regional state, northwest Ethiopia: a multi-level analysis	BMC Medical Informatics and Decision Making
Khan et al. [37]	2019	Bangladesh’s digital health journey: reflections on a decade of quiet revolution	WHO South East Asia J Public Health
Klungland [38]	2011	The Implementation of the District Health Information System in Mtwara and Lindi Regions in Tanzania	MSc Thesis UiO
Kossi et al. [39]	2013	Developing decentralised health information systems in developing countries–cases from Sierra Leone and Kenya	The Journal of Community Informatics
Mboera et al. [40]	2021	Data utilisation and factors influencing the performance of the health management information system in Tanzania	BMC Health Services Research
Moyo [41]	2017	Transformational Feedback: Breaking the vicious cycle of information use in Health Information Systems—A case from Malawi	PhD Thesis UiO
Nagbe et al. [42]	2019	Integrated disease surveillance and response implementation in Liberia, findings from a data quality audit, 2017	Pan Afr Med J
Nguyen & Nielsen [43]	2017	From Routine to Revolt: Improving Routine Health Data Quality and Relevance by Making Them Public	14^th^ IFIP 9.4 WG Conference
Nicol et al. [16]	2017	Perceptions about data-informed decisions: an assessment of information-use in high HIV-prevalence settings in South Africa	BMC Health Services Research
Odei-Lartey et al. [25]	2020	Utilization of the national cluster of district health information system for health service decision-making at the district, sub-district and community levels in selected districts of the Brong Ahafo region in Ghana	BMC Health Services Research
Ogega [44]	2017	Data use challenges and the potential of live data visualization tools: A case study of health data-use workshops in Zambia	MSc Thesis UiO
Ohiri et al. [45]	2016	An Assessment of Data Availability, Quality, and Use in Malaria Program Decision Making in Nigeria	Health Systems & Reform
Vaidyanathan & Sahay [46]	2015	Using Health Management Information for Action: A Historical Analysis of Tamil Nadu, India	13th IFIP 9.4 WG Conference
Vila-Pozo & Sahay [47]	2019	Institutional Shaping of Affordances: Implications on Information Use in Global Humanitarian Organizations	15^th^ IFIP 9.4 WG Conference

**Fig. 3:**
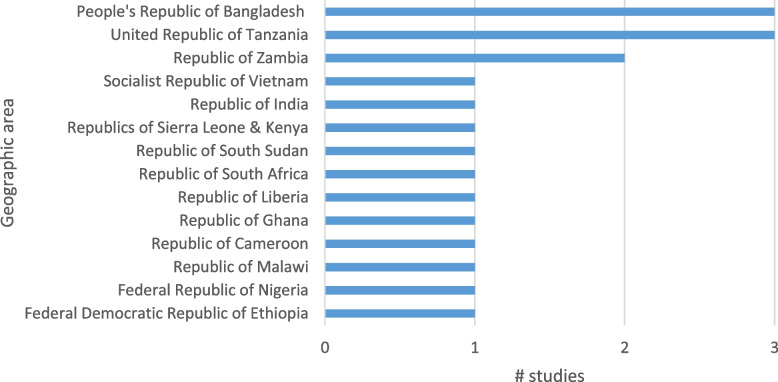
Geographic focus of studies

The included studies were predominantly from Africa (with one comparing two African countries) and the remaining studies were conducted in Asia. This is unsurprising given that DHIS2 has been primarily adopted as the routine HMIS in these two regions (see Fig. 3).

**Fig. 4:**
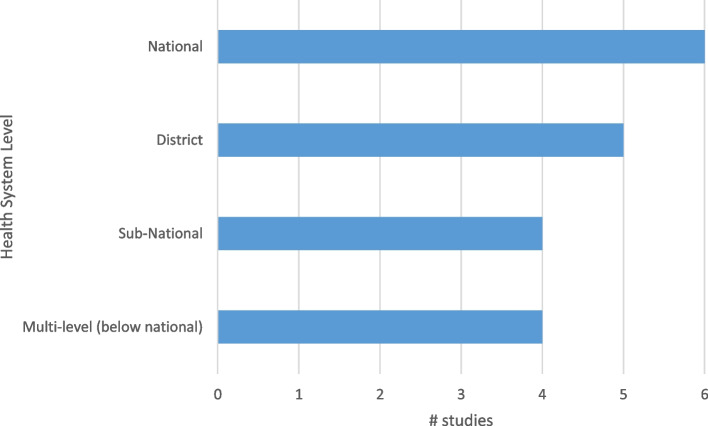
Level within the health system

There was a variety of levels within the health systems under investigation. There was a fairly even spread across national, sub-national, or district levels, and also in studies exploring different levels in the health system (see Fig. 4).

**Fig. 5:**
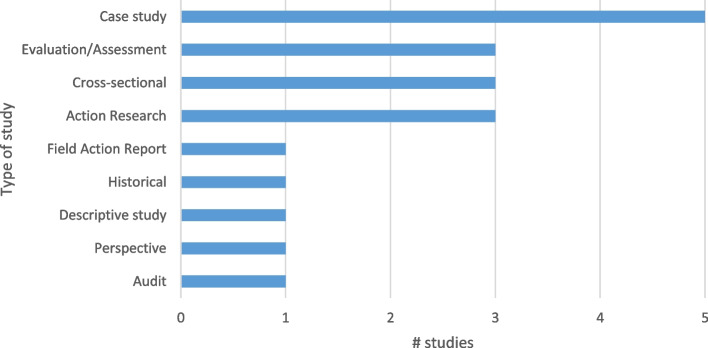
Type of study

Different programmatic areas were investigated with most of the studies focusing on HMIS in general (10). There was a wide range of other programmes in the remaining articles: Accident and Emergency (1), Community (2), Humanitarian (1), Integrated Disease Surveillance and Response (1), Malaria (1), HIV (1) and Maternal, Neonatal and Child Health (2). Case studies formed the basis of the most common study though there was quite a variety of study types as well as a mix of quantitative and qualitative data collection methods used (see Fig. 5).

### Categories of DHIS2 data use reported

A number of the reviewed articles that occasionally included the term’data use’ only mentioned that data was used or not used, or mentioned a desire to improve data use, or that data was being analysed, or mentioned use as being reported by people without any evidence or further description of use or how data were obtained. These documents were excluded at the full text review stage as they did not meet inclusion criteria.

Though there was some evidence that DHIS2 data were being used for evaluation of interventions, most of these reviews were not done routinely nor carried out by staff at an operational level within the health system. In many cases these studies were research studies testing a model or concept. For this reason, they were excluded from this review after full text review. Some examples included:

- Assessing technical inefficiency of hospitals [48].
- Evaluating indoor residual spraying in addition to long lasting insecticide-treated nets [49].
- Comparing effect of Performance Based Financing (PBF) and decentralized facility financing (DFF) on health service delivery [50].
- Determining the effectiveness of a national mass distribution campaign of long-lasting insecticide-treated nets & indoor residual spraying on clinical malaria [51].

Most of the reported examples in the articles included at the extraction stage covered a mixture of process, content, and purpose of routine data use.

#### Process

In many of the reviewed documents different information ‘products’ were cited, such as reports, dashboards, displaying of charts, and health bulletins. However, both the process by which these outputs were generated, and their end purpose, are largely absent except in cases where the reports were subsequently shared and compared in review meetings (see Purpose section). Nagbe et al. [42] to some extent connect the data analysis with the action by noting that '78% of the health facilities used bar chart as methods to detect outbreaks; 56% used trend lines; 33% used summary table and 11% used map to determine outbreaks’ ([52], p.4). Mboera et al. [40] note in their study in Tanzania that very few facilities (less than 10%) conduct proper analysis and display data by plotting graphs to illustrate disease burden. The most common data displayed on the wall were lists of top-ten diseases (58%), but most of these displays omitted dates, statistics or comparisons. At district level the facility data were mainly used to compile annual reports [40]. One example of another type of output was the use of maps to identify ‘death spots’ [35] at sub-districts level thus assisting health managers to concentrate necessary interventions.

Often the people who entered the data were not the same people who generated the products—in some cases statisticians or ‘IT’ personnel were assigned to tabulate the data and share the generated summary reports with district and divisional health managers [33, 38]. In other cases, perception or culture determined that facility staff were not key players in terms of using data for decision making: ‘the use of data in decision making is generally limited at national and sub national levels’ [53] and ‘district managers are not involved in decision-making as this is the responsibility of those at the central level’ [32]. However, Asah et al. [32] note that nurses claimed they used data to inform planning, but there was confusion over what constituted an indicator. Ogega [44] notes that whereas district staff were conversant with DHIS2 and the creation of dashboards and graphs, facility staff had to ask district staff to produce reports due to their limited access to DHIS2.

#### Content

The studies covered a wide variety of programmes and different levels in the health system. In regard to data use most of the studies showed that data were collected at a facility level and sent up to be compiled as quarterly reports which occasionally formed part of a review meeting or workshop. However, there was little detail on what indicators were compiled or what was contained in the reports. One example of a slightly more detailed description included: ‘The project thus initiated the use of quarterly bulletins, a modest four-page leaflet comparing all districts on a handful of health indicators, as well as some indicators on data quality’ [39]. This was accompanied by a photograph of the cover of one leaflet.

Some of the studies included information on how authors determined whether data was used, but details were lacking. For example, Chanyalew et al. [36] indicate that some comparative indicators were used: ‘Of the total 386 departments investigated, 200 (52%) calculated targets against achievement, and only 130 (33.7%) provided feedback to lower-level health workers. 50.3% had identified key indicators, 45.9% had health coverage calculated and 40.4% had decisions made on available information. As a result, the study revealed that only 46.9% of department heads utilized routine health information for evidence-based decisions.’ However, what was not indicated in the study was how health coverage was calculated and for which programme.

There were a couple of articles that showed content being produced for the general public. Nguyen and Nielsen [43] describe the publication of accident numbers during a particular festive period to raise awareness of the prevalence of accidents. Some articles included comments on making data more public [46], but exact details on how this was to be done were not described.

#### Purpose

In relation to Nutley’s categories on the purpose of data use [4] there were only two examples of *advocacy and policy development*. As noted above Nguyen and Nielsen [43] described the strategy whereby ‘Accident and Emergency’ attendances at hospitals were reported publicly in order to increase awareness of the number of accidents during a particular festival period. This approach resulted in the triangulation of data from the Ministry of Public Security and the Ministry of Health. This led to the process of querying differences in data and to different ministerial departments setting up meetings to validate the published data.

Odei-Lartey et al. [25] in their review of use of DHIS2 data in Ghana concluded they were being used to advocate for more resources and the formation/revision of policies/strategies (at a District Government Hospital, not at facility or community level). However, no details regarding how this was done were provided.

There were many examples of the use of DHIS2 data in terms of *program review and planning*. Ohiri et al. [45] mentioned that DHIS data were reportedly used most often for performance and/or supply chain management, but no further details were supplied. Data were most commonly exploited for developing periodic plans, monitoring and comparison of performance, review meetings, and reports (See Table 2).

**Table 2:** Examples of use of data for programme review and planning

**Planning**	Performance-based business planning (Asah et al. 2017) [32] National level evaluated programs when preparing annual report (Asah et al. 2017) [32] Visualise live data online- assists local planning, such as using death spot maps for interventions (Biswas 2017) [35] Development and implementation of district and zonal action plans (Moyo 2016) [53] Brief mention of data being used to detect outbreaks & data informing topics for health talks (Nagbe et al. 2019) [42] Shifting/mobilization of resources (Odei-Lartey et al. 2020) [25] District level use of HMIS for annual planning (Mboera et al., 2021) [40]
**Performance**	Performance monitoring at facility level with performance monitoring team discussions (Chanyalew er al 2021) [36] Scoring health facility performance (using DHIS2 and HRIS with a physical visit and patient satisfaction) (Khan et al. 2019) [37] League tables (using Excel software with DHIS2 data) and Certificates of Improvement (Kossi et al. 2013) [39] Performance improvement and sharing experiences with others at peer review meetings (Moyo, 2016) [53] HMIS league tables but feedback on how to improve ranking is poor (Vaidyanathan et al. 2015) [46] Performance recognition and role/responsibility revision (Odei-Lartey et al. 2020) [25] Facilities compared performance between service coverage, determining disease trends over time, and community health education and promotion (Mboera et al. 2021) [40]
**Reports**	Compiled quarterly reports at district level comparing results against targets (Asah et al. 2017) [32]
**Review meetings**	Monthly validation and review meetings sub-district, district and division levels (Begum et al. 2020) [33] Monthly feedback meetings at the district and national levels (Begum et al. 2020) [33] Quarterly data use workshops over 5 days and peer presentations at district level (Braa et al. 2012) [9] Peer review meetings/Information meetings over 2 days (Ogega, 2017) [44]

With respect to planning very little detail was given on how the DHIS2 informed the plans—in most documents there were simple statements about DHIS2 data informing plans and, in one case, excerpts from the plan were presented [32]. However, no additional detail was provided on how action plans were previously used, or not used, or on their future implementation. There was somewhat more detail supplied in regard to performance monitoring and comparison, with some detailed descriptions of league tables [39, 53], the recording of the discussions of performance monitoring teams [36], the scoring of health facility performance (using DHIS2 data with other data) [54] and HMIS league tables [46]. Mboera et al. [40] report that HMIS focal persons from 9 districts claimed that allocation of resources to facilities was based on good performance, but only two of those districts were able to provide evidence of any performance improvement tools used to monitor this good performance. Overall, little detail was provided about how performance monitoring was conducted (in terms of indicators used to assess performance), or about the stakeholders conducting the monitoring and the process by which feedback, if any, was given. Kossi et al. [39] include examples of certificates of improvement presented at the review meetings in Sierra Leone. It is stated that performance improved as a result of these comparisons but very few examples are given of specific actions undertaken.

More detail is provided on data review meetings and workshops. In a number of the examples presented there were also detailed descriptions of comparisons of performance and/or peer feedback on presentations. Braa et al. [9] describe in detail a 5-day data-use workshop, how the workshop was conducted as well as a detailed list of actions and changes made in the planning and delivery of health services across a number of programmes. Begum [33] describes, at different levels of the health system, review meetings that support the strengthening of the HIS. A District Information Meeting (DIM) in Rufunsa District in Zambia is also described in detail by Ogega [44] though the author outlines the absence of a standard process or format to these meetings, noting that ‘all that is being done is holding DIMs for procedural and formality purposes’ ([44], p.38).

Under Nutley’s third category of data use for *decision-making processes,* Odei-Lartey et al. [25] in their review categorised their findings on decision-making, centred on the taking of action-oriented decisions based on routine HMIS data, on discussions about the HMIS platform, and on actions taken to promote the usage of the HMIS system. However, in all of these decision-making processes, they found limited evidence of data use.

Chanyalew et al. [36] measured the proportion of routine health information utilisation using five core indicators: (i) presence of feedback provided by department heads to health workers in the department, (ii) evidence on the use of information for decision making, (iii) key performance indicators, (iv) evidence on health coverage, and (v) target achievements. They reported that the proportion of information use among department heads for decision making was estimated at 46%. However, this was based on responses from health facility department heads using a collection tool developed by the Performance of Routine Information System Management (PRISM) and not based on observation or validated with documentation.

A number of reviewed documents mention that action was taken based on DHIS2 data. For example, Ogega [44] notes that in review meetings performances are discussed, action plans are drawn up and a commitment to the plans is agreed. However, there is no discussion as to whether these plans are reviewed at subsequent meetings nor is there any description of the type of action that is included. Kossi et al. [39] note that chiefs took action based on league tables at chiefdom level and provide examples of improvements made in terms of institutional deliveries, e.g. local councillors passing bye-laws allowing for traditional Birth Attendants to assist pregnant women to have a clean and safe delivery in the health facility with training staff in attendance. After the review meetings and following requests by community leaders (Paramount chiefs), the District Health Management Team also organised outreach activities to increase the coverage for key health outcomes like childhood immunization. By asking questions in interviews Ohiri et al. [45] explored the use of data in relation to priority setting, surveillance, performance management, supply chain management, and advocacy and concluded that “DHIS data were reportedly used most often for performance and/or supply chain management” ([45], p.319). No examples are given.

In overall terms, in Table 3, examples of decisions made are included and illustrated. The most explicit account of actions and decisions made is in Braa et al. [9], where they provide a number of examples of improved data use resulting from their long term project for strengthening the HMIS in Zanzibar, the United Republic of Tanzania, 2005–2008.

**Table 3:** Examples of use of DHIS2 data for decision-making

Author	Decision making processes
Biemba et al. 2017 [34]	CHWs use mobile application to: send weekly reports to health centre supervisors on disease caseloads and medical commodities consumed, to make drug and supply requisitions, and to send pre-referral notices to health centres
Biswas 2017 [35]	Verbal autopsies used by local health managers for effective planning and reduction of such deaths in the future leading to: improvements in 1st delay (decision making) Improvements in 2nd delays (transferring to referral centre) and improvement in referrals
Braa et al. 2012 [9]	– Development of indicators to monitor emergency obstetric and neonatal care availability – Monitoring of quality of antenatal care and skilled birth attendance coverage – Introduction of maternal death audits – Introduction of the “couple year protection rate” indicator – Improved anaemia diagnosis in pregnancy Malaria Programme – Increased emphasis on bed net coverage – Monitoring of malaria in pregnancy – Treatment of confirmed rather than clinical cases, which in some instances resulted in data showing lower malaria incidence – Investigation of high dropout rates and coverage over 100% – Identification of double counting, resulting in improved quality control mechanisms – Introduction of diagnostic criteria to reduce misdiagnosis of pneumonia and malaria – Reduction of excessive data categories and age groupings – Routine collection of basic inpatient indicators such as average length of stay and bed occupancy rate – Focus on signal functions of emergency obstetric care and referrals, not just reporting of complications – Inclusion of laboratory data to check quality of diagnosis, particularly of malaria, tuberculosis, anaemia and syphilis – Improvement of OPD reporting to gain a more comprehensive idea of district-wide disease burden – Development of workload indicators to rationalize staffing needs and advocate for redistribution of staff away from central hospitals

## Discussion

Varying conceptualisations of data use are evident in all the documents reviewed. There is the medical focus on the clinical encounter in terms of tracking patients and managing cases (curative), the engineering perspective involving the manipulation of data into ‘usable’ formats (e.g., dashboards), and the public health perspective involving the use of data for disease prevention and health promotion. The latter category best fits the definition used in our scoping review, in terms of how data are routinely used to improve health care and service delivery at Primary Health Care levels. With this in mind, we concluded that many documents describing the use of data to generate charts and reports were not in fact providing examples of ‘data use in practice’ (unless there was a description of how those charts or reports were used, or a description of the process involved in their production).

It is also clear that many varying conceptualisations of the purpose, or type, of action expected from data are embedded in the HMIS. For example, Kelly et al. [55] question the more scientific ‘decisionistic’ focus on decision-making with an underlying ‘control at a distance’ ethos, i.e. using data in order to ‘control’ or manage performance of facilities, as opposed to processing data in order to provide occasions to hold ‘conversations that matter’. There is also recognition that evidence is socially and historically constructed—with different contexts different people will interpret evidence differently – a point made by Jones in his questioning of the assumptions underlying what is meant by data [20]. Related to this, in their review of design differences across their partnerships, Mutale et al. [56] conclude that different theories of change lead to different perceptions on what information is needed, on the manner in which that change is expected to take place and on who will be the users of that information. Madon et al. [57] also argue that there is a requirement to design and implement health information systems for local decision-making and accountability rather than reduce them to ‘mere reporting tools’. The view of HMIS as mechanisms for reporting is typical of centralistic attitudes to public sector management (see for example [58, 59]), and of expectations experienced in partnerships with international organisations. Each HMIS thus gives what Jones terms ‘a selective representation of the situation’ [20].

The shift over time over what constitutes evidence colours current debate on the use of data for action taking or decision making. Though clinical trials and other evidence for clinical decision making and delivery of health care are important sources of evidence, a more inclusive and sophisticated view of evidence has emerged (for example [60–63]), with the term evidence- ‘informed’ practice now in more widespread use than evidence- ‘based’ practice [64, 65]. It is questionable whether or not the concept of ‘data use’ is best suited to what we have defined as ‘data use’ in th is review, whether or not we should promote evidence informed action-taking and decision-making and adopt a different language to describe this.

Another debate arose in the work of Asah et al. [32] over who is expected to make decisions. In their case of Cameroon Asah et al. [32] note that decisions are not expected to be made at the level below district and, consequently, facility level users of the system only have permission to input data. Thus, requests for information are made via the district office due, firstly, to lack of access and, secondly, to the expectation that data will not influence decision-making or action. Wickremasinghe et al. [13] note that when the data collectors and users are separate entities it is safe to conclude that the system has been designed for monitoring rather than decision-making. Similarly, many instances of DHIS2 focus on reporting data upwards and not on the creation of data for use at operational or facility level – what Madon et al. [57] refer to as data being used ‘as reporting tools’, the end goal being a fixation on quality-reporting rather than on local use. Data quality and reporting rates are much easier to measure, and consequently easier for donors and governments alike to monitor and include in their evaluations and reports – to paraphrase Robert Chambers what gets measured counts [66], and gets done.

The theory of change in relation to data collection, its processing and use, is often simplistic. Even though there is now increased recognition of a more holistic approach that embraces technical, behavioural, and environmental/organisational aspects of data use, the main focus in documented HIS interventions remains focused on challenges faced or on technical solutions. Hoxha et al. [67] systematically reviewed technical, behavioural and organisational/environmental challenges that hinder the use of routine health information systems (RHIS) data in LMICs and the strategies implemented to overcome these challenges. They concluded that “Additional research is needed to identify effective strategies for addressing the determinants of RHIS use, particularly given the disconnect identified between the type of challenge most commonly described in the literature and the type of challenge most commonly targeted for interventions.” Of the studies identified in their review, the number of articles describing challenges to the use of RHIS was double that of studies describing strategies to overcome them. Additionally, they discovered that even though technical challenges were the least commonly raised challenges in the literature, strategies that incorporated technical components were the most prevalent, many of which involved a focus on developing indicators, registers, and tools for the improvement of data use. On the other hand, only 13% of RHIS strategies address organisational or environmental challenges such as resource shortages, training, feedback, and management even though more than half of the studies described these as challenges. Their review included DHIS2 interventions. So, though it is acknowledged that technology cannot be the sole driver for improved use but can be used as a catalyst for change, there remains a disproportionate focus (or, at least, a documented focus) on the technical side of enabling data use. As Noir and Walsham remind us – Information and Communication Technologies in health often play a ‘mythical and ceremonial role’ and do not necessarily constitute a means to support local action and decision making [68].

Overall, though, we do not conclude that routine data is not being used nor that there is an absence of data culture at facility level. Dahal [69] presents an interesting case. It illustrates that data is being used at the operational level by healthcare workers on a routine basis, but that this is a manual system. The routine data is sent up manually in the system to be included in DHIS2, but this data is never reported or fed back so is not available at lower level for use. There are also many examples of charts being presented on walls or in notebooks which are used to track performance and cases but are not based on the data that have been entered into DHIS2. For instance, Damtew et al. [70] report the case of a community health worker in South Africa who drew a map of the areas where all the tuberculosis patients lived, so that the staff could go and follow-up if patients did not show up for treatment. Likewise, Health Extension Workers in Ethiopia use hand drawn maps to plan daily activities. Similar community level data collection is reported by Moyo [41] in Malawi. However, in all these cases these data were not entered into DHIS2. This could be identified as a shortcoming of this review (see below), that by focusing solely on DHIS2 data it excludes other parallel data use practises. More importantly, though, it raises the question as to why such data are not being used within DHIS2 when there is the functionality within DHIS2 to do so. As Jones [20] notes there are costs to data—costs of producing, storing, retrieving and using data, and we need to consider these when investigating use. Chrysantina et al. [71] offer one possible explanation to non-use: we often assume health staff, once trained, know how to use the various functions in DHIS2, but they find that data literacy is a neglected area in medical school training and in the Continuing Professional Development element of the DHIS2 training curriculum. Walsham [7] notes the relevancy of Gigler’s [72] work on the different capabilities required to use the internet to improve well-being in an under-resourced community in Bolivia. Besides having basic IT capabilities three groups of informational capabilities are needed – communication, information literacy and knowledge sharing. Asah [73] investigates the role of facility managers in empowering the staff with such informational capabilities. Returning to how we conceptualise data Jones [20] argues that we need to understand how data came to be (in terms of what is considered to be the phenomenon, what are considered to be the data about the phenomenon, what can be recorded, what gets chosen to be recorded and what actually gets recorded) as well as how are data used (what gets looked at, what gets found, what gets extracted, what gets understood and what actually gets used). Fundamentally he argues that data in practice is a culmination of a long series of steps and at each step there is the possibility of breakdown and alteration of the data. It is perhaps a combination of the solutions suggested by the above authors that we need to explore.

Walsham’s [7] conclusions (to his reflection on information for action) summarise our discussion well. He raises 4 points:

- ICTs play a crucial role in improving data use but must be part of a more holistic approach that encompasses the technological, social, and institutional domains
- Capacity on data use for health workers requires strengthening
- Software development must be integrated with work practices and computerised systems of healthcare workers
- Institutional change is required to place greater emphasis on local accountability and empowerment

There are a number of limitations to this study. As mentioned earlier, the focus on DHIS2 may mean we have missed some examples of documented data use practice but given that DHIS2 is one of the largest routine HMIS the findings are likely to be applicable to other systems. The search keyword ‘DHIS’ may have resulted in missed articles or documents that do not specifically include the software platform name in the article, or may have excluded documents that have another name for their RHIS which is built on DHIS2. We tried to address this through the sharing of the draft scoping review article with forums involved in DHIS2 as mentioned, and to snowball from the reference lists of articles included. Additionally, one of the authors (JS) has worked with HISP for 20 years and, 15 years ago, the other author (EB) worked with HISP for a number of years, and has joined HISP as a guest researcher for the year. As interpretive researchers we acknowledge that this scoping review is conducted from a more internal perspective of HISP and that other perspectives and interpretations of the findings would also exist.

## Conclusion and implications for practice and research gaps

Overall, in response to our primary research question ‘How is DHIS2 data being used routinely for action and decision making within the health system?’ we can conclude that DHIS2 data is being used but there are few detailed descriptions of this use in peer review or grey literature. In one regard, this is surprising given the extent of the scale of implementation and use of DHIS2 and the many anecdotal stories we in HISP have of data use – it is in fact HISP’s *raison d’être*. On the other hand, the paucity of detailed descriptions of DHIS2 use is not surprising given that most of the literature on DHIS2 is written by HISP-affiliated people, who are increasingly occupied with making, implementing, and scaling software. A gap has grown steadily between use and technology development. An effect of the increased collaboration with international agencies is that HISP has become more and more focused on technology and software development rather than on an information system network as evidenced by the DHIS2 papers included in the review by Hoxha et al. [67] – a focus on software use rather than on data use.

Most commonly, we observe that DHIS2 data are being used primarily for planning and performance, for decision making and action taken. Whilst there are some rich descriptions in the examples provided, this richness could be enhanced if the use cases presented in these documents were made useful to other contexts. A three-pronged approach to improving data use is provided by Sahay et al. [74] based on considerable case material from India and Africa. They argue that we need to start focusing on conversations around data, supporting communities of practice as enablers of such conversations, and integrating IT solutions into the work processes and practices of the frontline healthcare worker. We would add to this that there is the need to document in more detail and share how data are being used. This may require research into why this documentation and dissemination is not currently being done. Further retrospective investigation into how data were created and who used the data could help make more explicit the philosophy behind the creation of HMIS, as well as the different conceptualisations of data, use and data use embedded in the HMIS.

## Supplementary Information

**Additional file 1: Appendix 1. **Search strings and databases used (up to March 22nd 2022.

## Data Availability

All data generated or analysed during this study are included in this published article.

## Abbreviations

DHIS2: District Health Information Software 2
HISP: Health Information Systems Programme
HMIS: Health Management Information Systems
IFIP: International Federation for Information Processing
IFIP9.4: International Federation for Information Processing: Working Group 9.4
LMICs: Lower and Middle Income Countries
PRISM: Performance of Routine Information System Management
RHIS: Routine Health Information Systems
UiO: University of Oslo

## Footnotes

1 Deliberate misreporting may occur to present a distorted picture of achievements which could take place to indicate the meeting of targets which may be above actual performance. Supervisors or line managers may require junior staff to deliberately misreport these data (7).

2 See Dhis2.org.
