# Supplementary material for: Routine use of DHIS2 data: a scoping review
Source: BMC Health Serv Res. 2022 Oct 6;22:1234. doi: 10.1186/s12913-022-08598-8 (PMC9535952; doi:10.1186/s12913-022-08598-8)
Supplement: Supplementary file 1 — Additional file 1: Appendix 1. Search strings and databases used (up to March 22nd 2022. [file 12913_2022_8598_MOESM1_ESM.docx]

**APPENDIX 1: Search strings and databases used (up to March 22nd 2022)**

**PubMed (n=79)**

**Search string: (“district health information system 2”) OR dhis2 OR dhis1 AND (“data use”) OR use OR utilisation Or utilization**

**Note “district health information system 1” was not found in the search so was removed**

("district health information system 2"[All Fields] OR "dhis2"[All Fields] OR "dhis1"[All Fields]) AND ("data use"[All Fields] OR ("statistics and numerical data"[MeSH Subheading] OR ("statistics"[All Fields] AND "numerical"[All Fields] AND "data"[All Fields]) OR "statistics and numerical data"[All Fields] OR "use"[All Fields]) OR (("statistics and numerical data"[MeSH Subheading] OR ("statistics"[All Fields] AND "numerical"[All Fields] AND "data"[All Fields]) OR "statistics and numerical data"[All Fields] OR "utilization"[All Fields] OR "utilisation"[All Fields] OR "utilisations"[All Fields] OR "utilise"[All Fields] OR "utilised"[All Fields] OR "utilises"[All Fields] OR "utilising"[All Fields] OR "utilities"[All Fields] OR "utility"[All Fields] OR "utilizations"[All Fields] OR "utilize"[All Fields] OR "utilized"[All Fields] OR "utilizer"[All Fields] OR "utilizers"[All Fields] OR "utilizes"[All Fields] OR "utilizing"[All Fields]) AND "Or"[All Fields] AND ("statistics and numerical data"[MeSH Subheading] OR ("statistics"[All Fields] AND "numerical"[All Fields] AND "data"[All Fields]) OR "statistics and numerical data"[All Fields] OR "utilization"[All Fields] OR "utilisation"[All Fields] OR "utilisations"[All Fields] OR "utilise"[All Fields] OR "utilised"[All Fields] OR "utilises"[All Fields] OR "utilising"[All Fields] OR "utilities"[All Fields] OR "utility"[All Fields] OR "utilizations"[All Fields] OR "utilize"[All Fields] OR "utilized"[All Fields] OR "utilizer"[All Fields] OR "utilizers"[All Fields] OR "utilizes"[All Fields] OR "utilizing"[All Fields])))

Translations

use: "statistics and numerical data"[Subheading] OR ("statistics"[All Fields] AND "numerical"[All Fields] AND "data"[All Fields]) OR "statistics and numerical data"[All Fields] OR "use"[All Fields]

utilisation: "statistics and numerical data"[Subheading] OR ("statistics"[All Fields] AND "numerical"[All Fields] AND "data"[All Fields]) OR "statistics and numerical data"[All Fields] OR "utilization"[All Fields] OR "utilisation"[All Fields] OR "utilisations"[All Fields] OR "utilise"[All Fields] OR "utilised"[All Fields] OR "utilises"[All Fields] OR "utilising"[All Fields] OR "utilities"[All Fields] OR "utility"[All Fields] OR "utilizations"[All Fields] OR "utilize"[All Fields] OR "utilized"[All Fields] OR "utilizer"[All Fields] OR "utilizers"[All Fields] OR "utilizes"[All Fields] OR "utilizing"[All Fields]

utilization: "statistics and numerical data"[Subheading] OR ("statistics"[All Fields] AND "numerical"[All Fields] AND "data"[All Fields]) OR "statistics and numerical data"[All Fields] OR "utilization"[All Fields] OR "utilisation"[All Fields] OR "utilisations"[All Fields] OR "utilise"[All Fields] OR "utilised"[All Fields] OR "utilises"[All Fields] OR "utilising"[All Fields] OR "utilities"[All Fields] OR "utility"[All Fields] OR "utilizations"[All Fields] OR "utilize"[All Fields] OR "utilized"[All Fields] OR "utilizer"[All Fields] OR "utilizers"[All Fields] OR "utilizes"[All Fields] OR "utilizing"[All Fields]

**Web of Science (n = 115)**

**(ALL=((“district health information system 2”) OR dhis2 OR (“district health information system 1”) OR dhis1)) AND ALL=((“data use”) OR use OR utilisation Or utilization)**

**Embase (n=90)**

**search string: ('district health information system 2') OR dhis2 OR ('district health information system' NEXT/1 2) AND ('data use') OR use OR utilisation Or utilization**
